# Incorporation of Mycelium (*Pleurotus eryngii*) in Pea Protein Based Low Moisture Meat Analogue: Effect on Its Physicochemical, Rehydration and Structural Properties

**DOI:** 10.3390/foods11162476

**Published:** 2022-08-17

**Authors:** Shubham Mandliya, Anubhav Pratap-Singh, Siddharth Vishwakarma, Chandrakant Genu Dalbhagat, Hari Niwas Mishra

**Affiliations:** 1 Agricultural and Food Engineering Department, Indian Institute of Technology Kharagpur, Kharagpur 721302, India; 2 Food Nutrition & Health Program, Faculty of Land & Food Systems, The University of British Columbia, Vancouver, BC V6T 1Z4, Canada

**Keywords:** mycelium, meat analogue, protein solubility, extrusion, alternative proteins

## Abstract

The protein content of a plant-based ingredient is generally lower than its animal food counterpart, and research into novel alternative protein is required that can provide similar protein content, texture and appearance as meat. This work investigates a mycelium-based low moisture meat analogue (LMMA) approach, by incorporating 0 to 40% *w*/*w* mycelium (MY) into pea protein isolate (PPI) via extrusion using a twin-screw extruder at 140 °C die temperature, 40 rpm screw speed, and 10 rpm feeder speed (0.53–0.54 kg/h). Physicochemical, rehydration, and structural properties of LMMA were assessed. The MY incorporation led to a significant change in color attributes due to Maillard reaction during extrusion. Water solubility index and water absorption capacity increased significantly with MY addition, owing to its porous structure. Oil absorption capacity increased due to increased hydrophobic interactions post-extrusion. Protein solubility decreased initially (upto 20% *w*/*w* MY), and increased afterwards, while the water holding capacity (WHC) and volumetric expansion ratio (VER) of LMMA enhanced with MY addition upto 30% *w*/*w*. Conversely, WHC and VER decreased for 40% *w*/*w* which was verified with the microstructure and FTIR analysis. Overall, MY (30% *w*/*w*) in PPI produced a fibrous and porous LMMA, showing future potential with an increasingly plant-based product market and decreasing carbon footprint of food production activities.

## 1. Introduction

Meat alternatives are restructured products that replicate processed meats in terms of texture, flavor, and appearance [1]. These products are in high demand due to the rising world population, limited natural resources, ethical, social and religious aspects of consuming animal meat, cost considerations and health problems [2]. They are mainly classified as plant-based, cell-based, and fermentation-based. However, they are mostly formed through texturized vegetable proteins which can replicate the fibrillar structure of meat muscle [3]. Various plant protein sources have been used for producing meat analogue, such as soy protein, wheat protein and pea protein.

Meat substitutes require rigorous processing such as thermo-extrusion, shear, spinning, and crosslinking, for the production of fibrous structures such as those of meat [1]. The former technique has high productivity, low cost, versatility, and energy efficiency with utilization in transforming native globules into filamentous aggregates or interactive fibers [4]. This technology, mainly low moisture extrusion, has the potential to form muscle-meat-like structure via the production of big pieces of texturized protein [5]. The more prominent product in the meat analogue category is the chunk-type meat analogue which is primarily produced from low-moisture extrusion cooking. This produce is a fibrous texture-based small-texturized protein pellet with the potential to rehydrate, and can be easily mixed with the ingredients necessary for the production of finished meat analogues [6].

Several studies have applied soy protein and wheat protein as a texturized vegetable protein to provide a texture, appearance, taste, smell, functionality, and nutrition similar to meat [7]. However, soy protein can be allergenic to some consumers, whereas the gluten in wheat protein can be problematic for celiac disease patients [8]. 

Pea protein is of high interest due to its high nutritional characteristics and low allergic responses compared with soy protein. Peas are high in essential amino acid lysine and branched-chain amino acids and are frequently used for texturization under low moisture conditions [9,10]. The utilization of pea protein produces meat analogue which, when rehydrated, has a meat-like fibrous texture with an absence of bitter taste [10]. The texture of the meat analogue developed from soya and wheat protein is tough, rubbery, and elastic, which produces poor mouthfeel [11].

Mycelium, as a novel alternative protein source, can be used to develop meat analogue with better texture consistency and good mouthfeel by using a fiber matrix, without any allergen problems [11]. It can be easily grown using submerged fermentation in 15–30 days with limited land and water resources [11,12], and is rich in protein, fiber, vitamin D_2_ and all the eight essential amino acids [12,13]. The filamentous mycelium protein can impart a meat-like chewable characteristic, and is also considered as beneficial to health [1]. Further, mycelium of *Pleurotus eryngii* species also has nutraceutical properties that can be utilized to form functional food helpful to fight against COVID-19 [12,14]. Meat analogue derived from mycelium has a meaty flavor due to the presence of high sulfur-containing amino acids and glutamic acid, implying a characteristic umami flavor [15].

Very limited work has been reported on meat analogue preparation from mycelium. Properties such as water holding capacity, protein solubility index and microstructure are highly important to determine regarding the quality of meat analogue. In this study, five formulations of mycelium and pea protein isolate were developed into low moisture meat analogues, and their physiochemical, rehydration, and microstructural properties, and secondary structure of proteins were analyzed.

## 2. Materials and Methods

### 2.1. Materials

The pea protein isolate (PPI) with 80% protein was obtained from MyFitFuel (New Delhi, India). Mycelium (MY) of *Pleurotus eryngii* species with 25% protein was acquired from the Indian Institute of Technology Delhi, Delhi [12]. Mycelium was pressed, freeze-dried, ground and sieved using 250 µm mesh and then stored in a deep freezer (−20 °C) until further use. The moisture content of the sieved PPI and MY were 0.097 and 0.098 gram water/gram dry matter, respectively [13]. All chemicals and reagents used in the experiments were of analytical/food grade.

### 2.2. Preparation of Meat Analogue

The MY was incorporated into the PPI using five formulations for the development of low moisture meat analogue (LMMA), with 100% PPI as the control. The five formulations were S0 (MY:PPI—0:100 *w*/*w*), S10 (MY:PPI—10:90 *w*/*w*), S20 (MY:PPI—20:80 *w*/*w*), S30 (MY:PPI—30:70 *w*/*w*) and S40 (MY:PPI—40:60 *w*/*w*). The samples were mixed and moisturized to 30% wet basis (wb) using a planetary mixer (Reico Equipment & Instrument, Kolkata, India) and refrigerated overnight for moisture equilibration. The protein content was measured using the Kjeldahl method [16]. The moisture and protein content of the mixture is shown in Table 1. The LMMA was prepared with the use of a co-rotating twin-screw extruder (KETSE 20/40, Brabender GmbH & Co.KG, Duisberg, Germany). The extrusion process parameters were kept constant for preparing all formulations and the extrusion process parameters were 140 °C die temperature, 40 rpm screw speed, and 10 rpm feeder speed, corresponding to a feed rate of 0.53–0.54 kg/h. A rectangular opening die (15 mm width × 5 mm height) was used to shape the LMMA. The torque, die pressure, and mass temperature were recorded at the end of screw (before the die), where the temperature was set at 110 °C [17]. The LMMA were dried using a recirculating hot air tray dryer (Basic Technology Pvt. Ltd., Kolkata, India) at 50 °C and 1 m/s air speed, for 6 h. Dried LMMA samples were packed in zip lock bags and kept at room temperature (25 °C) until analysis for rehydration and textural properties. The dried strips were ground, sieved (250 µm) and packed in zip lock bags until further analysis for physicochemical properties. All formulations were subjected to the same process treatment.

### 2.3. Physicochemical Properties

The physicochemical properties of the raw blends and their extruded counterpart were analyzed. The raw blends and extruded samples were ground and sieved into powders, for all properties except color.

#### 2.3.1. Color Characteristics

The color attributes were determined by colorimeter (CM-5, Konica Minolta, Tokyo, Japan). The total color difference (∆E) was calculated as described by Dalbhagat and Mishra [18] using Equation (1):

(1) $$\Delta E=\sqrt{{\left(L^{*}-L\right)}^{2}+{\left(a^{*}-a\right)}^{2}+{\left(b^{*}-b\right)}^{2}}\;$$

where, $L^{*},{\;a}^{*},{\;\mathrm{and}\;b}^{*}$ are the color parameters of formulation S0, and L, a, b are the color parameters of formulations S10 to S40.

#### 2.3.2. Water Solubility Index (WSI) 

Dried LMMA powder (1 g) was mixed with 10 mL distilled water and vortexed for 15 min, and then centrifuged at 4000 rpm for 20 min (C-24 Plus, Remi, Mumbai, India). The supernatant was collected in a pre-weighed Petri plate and oven-dried at 85 °C overnight. The dried plate was weighed, and WSI was evaluated using Equation (2):

(2) $$\mathrm{WSI}\;\left(\%\right)=\frac{m_{d}}{m}\times 100\;$$

where m_d_ is the dried supernatant weight, and m is the initial sample weight.

#### 2.3.3. Water Absorption Capacity (WAC) and Oil Absorption Capacity (OAC) 

WAC and OAC were measured according to the method described by Samard and Ryu [19], with slight modifications. Dried powder (1 g) was suspended in 10 mL distilled water in a pre-weighed centrifuge tube and vortexed for 15 min, and then centrifuged at 4000 rpm for 20 min. The OAC measurement was carried out using a similar procedure with refined soybean oil with a density of 0.91 kg/m^3^. The WAC and OAC were calculated using Equations (3) and (4):

(3) $$\mathrm{WAC}\;\left(\%\right)=\frac{m_{w}}{m}\times 100\;$$

(4) $$\mathrm{OAC}\;\left(\%\right)=\frac{m_{o}}{m}\times 100\;$$

where m, m_w_, and m_o_ are the initial sample weight, total weight of the moist sample, and total weight of the oiled sample, respectively.

### 2.4. Protein Solubility Index

The protein solubility index (PSI) was measured according to the method given by Morr et al. [20], with some modifications. Briefly, 200 g dried ground sample was mixed with 50 mL 0.1 N NaCl and vortexed for 10 min to form a paste. The pH of paste was measured, and Millipore water was added to make up a volume of 50 mL. The pH of the dispersions was adjusted to 7 using 0.1 N HCl and 0.1 N NaOH. The dispersed samples were vortexed again for 15 min, followed by centrifugation at 10,000 rpm for 20 min at 4 °C. The supernatant was collected using a muslin cloth, and the protein content of the supernatant was determined by the Kjeldahl method (UDK 159, Velp Scientifica, Usmate Velate, Italy) [16]. Similarly, the protein content measurement of the dried sample was carried out using the Kjeldahl method to calculate the protein solubility index.

### 2.5. Rehydration Properties

The dried LMMA samples used for rehydration were cut into uniform dimensions (15 × 10 × 5 mm). The water holding capacity (WHC), and volumetric expansion ratio (VER) were analyzed after the rehydration.

#### 2.5.1. Water Holding Capacity

The WHC was measured as described by Samard and Ryu [19] with slight modifications. The 5 g dried LMMA was rehydrated in 100 mL Millipore water and kept at 50 °C for 10 h, and then drained for 10 min. The WHC was evaluated using Equation (5):

(5) $$\mathrm{WHC}\;\left(\%\right)=\frac{m_{a}-m_{b}}{m_{b}}\times 100\;\;$$

where m_a_ and m_b_ are the weight after and before rehydration (g), respectively.

#### 2.5.2. Volumetric Expansion Ratio

The dimensions (L × W × H) of the LMMA were measured before and after the rehydration using a vernier caliper (Series 532–120, Mitutoyo Corp., Kawasaki, Japan) to calculate the volumetric expansion. The LMMA was considered to be a cuboidal shape in measuring the expansion ratio. The volumetric expansion ratio was calculated using Equation (6):

(6) $$\mathrm{VER}=\frac{\mathrm{volume}\;\mathrm{of}\;\mathrm{cuboid}\;\mathrm{after}\;\mathrm{rehydration}}{\mathrm{volume}\;\mathrm{of}\;\mathrm{cuboid}\;\mathrm{before}\;\mathrm{rehydration}}\;$$

### 2.6. Microstructure

Microstructure measurement of the LMMA was carried out using scanning electron microscopy (SEM) at a voltage of 5 kV (ZEISS EVO, Jena, Germany). The samples were rehydrated as described in Section 2.5.1, followed by freeze-drying at −50 °C. The dried samples were secured on studs and coated with a layer of gold–palladium (360 Å thick) under vacuum, followed by SEM. The images were taken at 5000× magnifications.

### 2.7. Secondary Structure of LMMA 

Fourier-transform infrared (FTIR) spectroscopy was used with a recorded frequency from 400 to 4000 cm^−1^ by FTIR spectrophotometer (Nicolet 6700, Thermo Fisher Scientific, Waltham, MA, USA) to analyze the PPI–MY based LMMA. Changes in secondary structure in protein are analyzed in meat analogues after extrusion, i.e., the amide I band (1600–1700 cm^−1^) and amide II band (1500–1600 cm^−1^) [21,22,23]. The ground samples were mixed with potassium bromide and a pellet was formed, which was hydraulically pressed, then placed in an IR beam. The amide II and amide I bands of FTIR spectra were deconvoluted by Peakfit software to analyze the secondary structure, and the Gaussian algorithm was used for peak fitting. The amide I bonds were observed and assigned from 1620 to 1640 cm^−1^ for β-sheets, 1650 to 1658 cm^−1^ for α-helix, 1662–1678 cm^−1^ for β-turns, and 1680–1695 cm^−1^ for antiparallel β-sheets [21,22,24].

### 2.8. Statistical Analysis

All the experiments were conducted in triplicate, and data are presented as mean ± standard deviation (SD). Tukey test was applied for the determination of significant terms (*p* ≤ 0.05) with Minitab 17.1.0 (Minitab LLC, State College, PA, USA). The FTIR data analysis was performed using Origin 18 (OriginLab Corporation, Northampton, MA, USA) and Peakfit 4.12 (Peakfit, SPSS Inc., Chicago, IL, USA).

## 3. Results and Discussion

The five formulations based on MY and PPI were subjected to constant extrusion conditions to prepare the LMMA. The thermomechanical treatment unraveled the protein through the interruption of hydrogen bonds and van der Waals’ forces, and developed a new crosslinking of amide bonds forming the matrix of the meat analogue [25]. The increase in concentration of MY significantly affected the physicochemical, rehydration, and secondary structure of protein and microstructure of LMMA. Detailed results and discussions are as follows.

### 3.1. Physicochemical Properties of Raw Blends and Extruded LMMA

#### 3.1.1. Color Characteristics

The effects of mycelium incorporation on the color characteristics of raw blend and dried LMMA are shown in Table 2. The lightness (L*) value decreased significantly (*p* < 0.05) with the addition of MY, except for extrudate S10. The decrease in lightness was due to the darker color of MY as seen in the raw blends. The extruded LMMAs were darker than the raw blends. This can be attributed to the Maillard reaction, with the interaction between amino acid and starch [5,26]. The b* value increased significantly (*p* < 0.05) for the raw blends due to the higher b* value of MY. The b* value decreased with the increase in mycelium content (20–40%), showing a decrement in yellow color. No significant change (*p* > 0.05) was found in the redness (a*) with mycelium increments from 10 to 40% in both raw and LMMA samples. The total color difference (ΔE) increased significantly (*p* < 0.05) with an increase in MY content for both the raw and LMMA samples. With 10% MY incorporation, ΔE was found at 1.19 (raw blend) and 1.21 (extruded LMMA) which was less than the human perception (ΔE = 2.2) [27] showing no significant change (*p* > 0.05) in the LMMA color attributes.

#### 3.1.2. Water Solubility Index 

**Figure 1:**
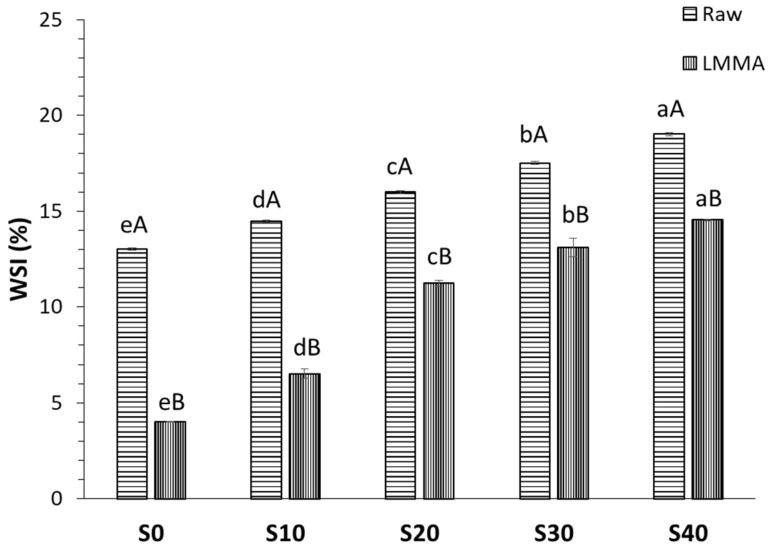
Effect of mycelium addition on water solubility index. All the values are mean ± SD (*n* = 3). Different capital letters in same formulation on the top of bar show significant difference (*p* < 0.05). Different lower case letters showed significant difference (*p* < 0.05) between the same type, i.e., raw or extruded.

The WSI evaluates the amount of water that can be extracted. The WSI of the raw blends and the dried LMMA samples are shown in Figure 1. The WSI increased significantly (*p* < 0.05) with the increase in MY content in the raw blends. The lowest WSI for sample S0 can be correlated with its higher protein content and weak functional properties [28]. Similar to raw blends, with MY addition, WSI increased significantly (*p* < 0.05), with the highest WSI (14.54 ± 0.02%) at 40% MY. This might be due to the higher water solubility of MY (27.83 ± 0.78%), as well as the decrease in protein content, thereby less protein denaturation during extrusion [29]. MY addition increased the carbohydrate content which, in turn, dextrinized during extrusion, resulting in a higher WSI value [29]. The WSI of the extruded sample was lower due to its dense structure. Similar results were reported by Kaleda et al. [10] in oat–pea protein blends and their meat analogues.

#### 3.1.3. Water Absorption Capacity and Oil Absorption Capacity 

**Figure 2:**
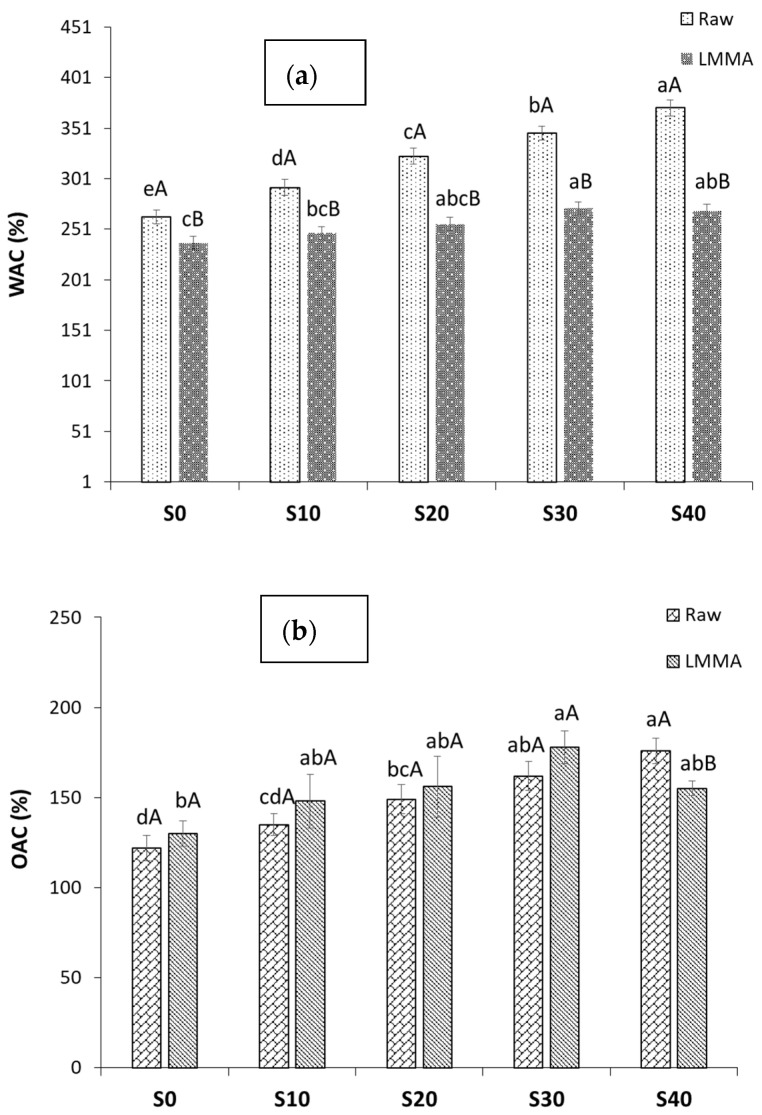
Effect of mycelium addition on (**a**) water absorption capacity, and (**b**) oil absorption capacity. All the values are mean ± SD (*n* = 3). Different capital letters in the same formulation on the top of bar showed significant difference (*p* < 0.05). Different lower case letters showed significant difference (*p* < 0.05) between same type, i.e., raw or extruded.

The WAC of the LMMA indicates its quality, preferably texture after rehydration, whereas the OAC indicates the flavor and mouthfeel by entrapment of oil [30]. The WAC and OAC of the raw blends and the effect of the addition of mycelium on PPI-based LMMA on WAC and OAC is presented in Figure 2a,b. Water binds the hydrophilic groups via hydrogen bonds, and oil binds the hydrophobic groups of the protein chains. The WAC and OAC of the raw blends increased significantly (*p* < 0.05) with the addition of MY in both raw as well as extruded samples. This was due to the fibrous and porous structure of mycelium which holds the water or oil in its pores [31]. The WAC of extruded LMMA was lower than its raw blend due to its dense microstructure and hard shell development during extrusion processing [10,13]. The WAC and OAC content of the LMMA ranged from 237.3% to 271.7%, and 130% to 178%, respectively. The higher WAC and low protein solubility may be attributed to the preservation of the protein network structure in the presence of mycelium [18]. The results of the WAC and OAC were in agreement with Samard and Ryu [18] and Lam et al. [32] for isolated pea protein. Similar results were found by Osen et al. [9].

### 3.2. Protein Solubility Index

**Figure 3:**
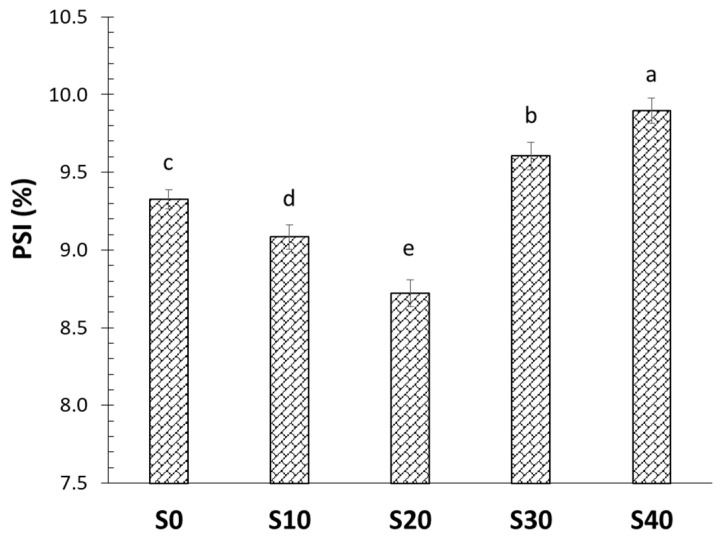
Effect of mycelium addition on protein solubility index at pH 7. All the values are mean ± SD (*n* = 3). Different letters on the top of bar show significant difference (*p* < 0.05).

Protein solubility indicates the texturization of protein as well as protein quality [33]. It can also be used to investigate the interaction between proteins from different sources. The effect of mycelium on the protein solubility index of PPI-based LMMA at pH 7 is shown in Figure 3. The PSI of S0, i.e., the control sample, was 9.33%, which was in agreement with Samard and Ryu [19]. The PSI was initially decreased (*p* < 0.05) until 20% mycelium addition, then afterwards it increased significantly (*p* < 0.05), showing the protein quality, denaturation and protein digestibility of the LMMA. The lower solubility in the S10 sample indicated higher denaturation of proteins that will eventually lead to higher protein digestibility and can lead to higher texturization of proteins [7,34]. An increase in fiber formation also resulted in a decrease in the protein solubility, however, after a certain increment in mycelium, the structure became less firm due to high fiber, and less interaction between protein–fiber and protein–protein resulted in an increase in PSI [35]. This can be seen from the low rehydration properties of the LMMA. 

### 3.3. Rehydration Properties

#### 3.3.1. Water Holding Capacity

WHC indicates the amount of water that can be bound by LMMA during rehydration and to form the protein gel network. WHC directly affects the juiciness of the LMMA. The effect of mycelium on the WHC of the rehydrated LMMA is shown in Table 3. The increase in MY decreased the free water for syneresis, resulting in a significant increase (*p* < 0.05) in WHC of LMMA. This may be attributed to the porous structure of the mycelium [31,36]. The cell structure and porosity highly affected the WHC [34]. However, the WHC at 40% mycelium was found to be the least, due to structure disintegration during rehydration. This data were in agreement with the microstructure.

#### 3.3.2. Volumetric Expansion Ratio

The volumetric expansion ratio indicates the expansion of the meat analogue during rehydration. The effect of mycelium on VER is shown in Table 3. Incremental increase in the mycelium content results in a significant increase (*p* < 0.05) in the VER, with the highest VER (4.14 ± 0.10) at 30% mycelium, followed by 20% mycelium. This was due to the increase in porous structure, as can be verified from the microstructure. Further increase in the mycelium content drastically decreased the VER. This may be attributed to the disintegration of the LMMA during rehydration.

### 3.4. Effect of Mycelium on Extrusion Process Parameters

The flow behaviour of the melt was generally described by the die pressure and torque [9,25]. The MY concentration had direct effect on the pressure and torque of the extruder. The pressure and torque of sample S0 with 0% MY concentration was 140.6 ± 2.3 bar and 24.15 ± 0.19 Nm. The addition of MY significantly (*p* < 0.05) decreased the pressure and torque to 99.3 ± 3.1 bar and 13.67 ± 0.27 Nm. This decrease in pressure and torque may be attributed to an increase in mass temperature (109 ± 1.5 °C to 129 ± 2.2 °C) and degradation of polysaccharides, thereby reducing the melt viscosity [37]. The increase in mass temperature was due to interparticulate friction, and friction between the feed particles and screw and/or extruder barrel [17].

### 3.5. Microstructure

**Figure 4:**
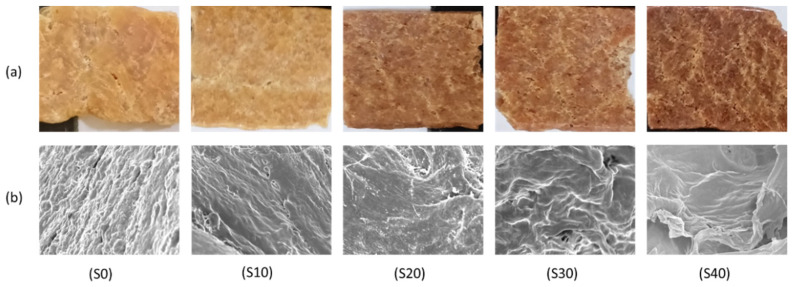
(**a**) Surface appearance and microstructure of the dried LMMA, and (**b**) freeze-dried rehydrated LMMA.

The surface morphology and the microstructure of the LMMA and freeze-dried rehydrated formulations are shown in Figure 4. The fibrous formation can be seen in Figure 4a, which can be validated with the microstructure in Figure 4b, where the addition of mycelium led to a porous structure. Similar results were obtained by Yuliarti et al. [38] in PPI. The densest structure was found in S0 formulation (100% PPI), which decreased with an increase in mycelium proportion. It was observed that a gradual increase in mycelium concentration led to a more fibrous and porous structure. The microstructure showed a rough structure due to the aggregated network [39]. The morphology of the formulations correlated with the WAC and WHC, as high porous structure leads to higher WAC and WHC, that will eventually lead to better reconstitution.

### 3.6. Secondary Structure of Proteins

**Figure 5:**
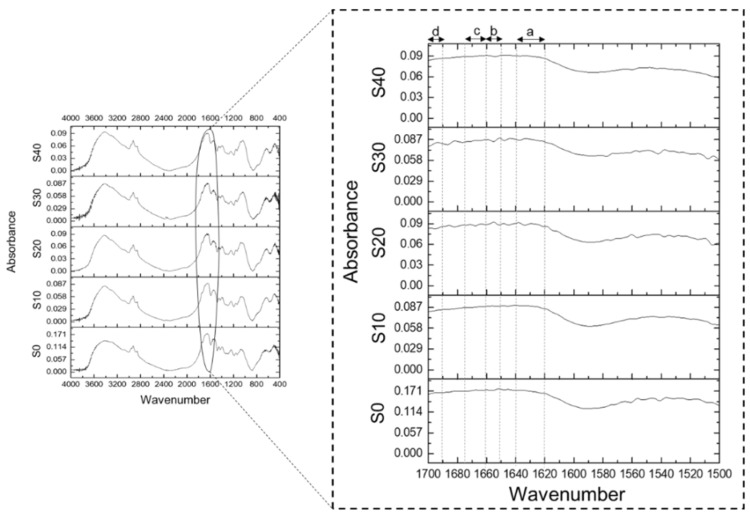
(a) FTIR spectra of MY–PPI blends LMMA in amide II and amide I regions. β-sheets, (b) α-helix, (c) β-turn and loops, and (d) antiparallel β-sheets.

In the processed LMMA, the effect of MY addition on the secondary structure change of proteins was explored. The FTIR spectra of the amide II (1500–1600 cm^−1^) and amide I (1600–1700 cm^−1^) bands for LMMA are shown in Figure 5. The amide II region indicates the bending of N–H bond and C–N stretching vibration showing less protein sensitivity against secondary structure, whereas the amide I region shows the stretch of C=O which is the most subtle area for studying the secondary structure of proteins [24]. It can be clearly observed that an increase in the MY proportion affects the secondary structure of the processed LMMA. Peaks were found in the range of 1620–1640 cm^−1^ for β-sheets (a), 1650–1659 cm^−1^ for α-helix (b), 1660–1675 for β-turn (c), and 1690–1695 cm^−1^ for antiparallel β-sheets (d). Similar outcomes were presented by Palanisamy et al. [21] for Spirulina/lupin-based meat analogue, and Beck et al. [22] for PPI. Other than the defined peaks, the peak near 1645 cm^−1^ represents random coil, and near 1663 cm^−1^ represents 3_10_ helix structure. The peak at amide II region may be due to the hydrogen bond formation between N–H and C=O [40]. For S0 sample, high absorbance well defined peaks with less noise, were observed. This may be attributed to the high protein concentration and low mineral components [41]. The bonds remain intact with the gradual increase in MY concentration. Addition of MY content does not cause any generation of a new absorbance peak, as seen from the deconvolated graphs of FTIR spectra (Figure S1).

## 4. Conclusions

The effect of mycelium on pea-protein-based meat analogue has been analysed through physicochemical, rehydration, and structural properties. The addition of MY resulted in browner LMMAs due to Maillard reaction. The addition of MY (up to 30%) increased WSI, WAC, OAC, WHC, and VER, along with better microstructure and secondary structure of proteins. Further increase in the MY concentration led to the disintegration of meat analogue during rehydration, thereby forming a loose structure resulting in a decrease in WAC, OAC, WHC, and VER, and an increase in PSI. Microstructure and secondary structure of protein showed favourable results for MY addition (30%). Overall, it can be concluded that up to 30% MY concentration has minimal effect on structure but enhances the quality parameters (WSI, WAC, OAC, WHC, VER, etc.) leading to a low moisture fibrous meat analogue. Further research is warranted to investigate other functional benefits that mycelium might produce when incorporated as a potential novel protein alternative in functional meat analogues. Moreover, the effect of extrusion process parameters needs to be researched, to understand the changes in the properties of LMMA.

## Figures and Tables

**Table 1:** Moisture and protein content of the samples before extrusion.

Sample	MY (% *w*/*w*)	PPI (% *w*/*w*)	Extrusion Moisture Content (% wb)	Protein Content (g/100 g)
S0	0	100	30	55.2 ± 1.2 ^a^
S10	10	90	30	51.5 ± 1.4 ^a^
S20	20	80	30	46.0 ± 1.9 ^b^
S30	30	70	30	41.2 ± 2.1 ^c^
S40	40	60	30	37.9 ± 0.9 ^c^

All values of protein are mean ± SD (*n* = 3). Different superscripts in the same column show significant difference (*p* < 0.05).

**Table 2:** Color characteristics of the raw blend and dried LMMA.

Sample	L*		a*		b*		ΔE	
	Raw	Extruded	Raw	Extruded	Raw	Extruded	Raw	Extruded
S0	75.49 ± 0.19 ^aA^	49.14 ± 0.65 ^aB^	5.69 ± 0.06 ^aB^	10.14 ± 0.13 ^aA^	24.91 ± 0.12 ^cA^	22.77 ± 0.64 ^aB^		
S10	74.31 ± 0.12 ^bA^	48.79 ± 0.92 ^aB^	5.62 ± 0.06 ^aB^	9.75 ± 0.18 ^aA^	24.82 ± 0.11 ^cA^	23.54 ± 0.39 ^aB^	1.19 ± 0.11 ^dA^	1.21 ± 0.33 ^dA^
S20	72.48 ± 0.25 ^cA^	44.13 ± 0.80 ^bB^	5.61 ± 0.03 ^aB^	9.95 ± 0.19 ^aA^	24.98 ± 0.10 ^bcA^	20.96 ± 0.34 ^bB^	3.01 ± 0.25 ^cB^	5.33 ± 0.85 ^cA^
S30	70.46 ± 0.33 ^dA^	41.00 ± 0.47 ^cB^	5.60 ± 0.07 ^aB^	10.18 ± 0.12 ^aA^	25.24 ± 0.06 ^bA^	18.23 ± 0.31 ^cB^	5.04 ± 0.34 ^bB^	9.32 ± 0.55 ^bA^
S40	68.49 ± 0.41 ^eA^	36.95 ± 1.08 ^dB^	5.80 ± 0.24 ^aB^	9.86 ± 0.22 ^aA^	25.92 ± 0.06 ^aA^	15.99 ± 1.06 ^dB^	7.07 ± 0.39 ^aB^	13.96 ± 1.43 ^aA^

All the values are mean ± SD (*n* = 3). Different superscripts in the same column (lower case letters) and in the same row (capital letters) show significant difference (*p* < 0.05).

**Table 3:** Rehydration properties of different formulations based LMMA.

Sample	WHC (%)	VER
S0	259.85 ± 19.18 ^c^	2.81 ± 0.20 ^c^
S10	385.28 ± 17.83 ^b^	3.45 ± 0.11 ^b^
S20	450.24 ± 22.29 ^a^	3.97 ± 0.22 ^ab^
S30	469.37 ± 16.14 ^a^	4.14 ± 0.10 ^a^
S40	192.51 ± 28.35 ^d^	2.62 ± 0.27 ^c^

All the values are mean ± SD (*n* = 3). Different superscripts in the same column show significant difference (*p* < 0.05).

## Data Availability

Data supporting the reported results are available upon request.

## Supplementary Materials

The following supporting information can be downloaded at: https://www.mdpi.com/article/10.3390/foods11162476/s1, Figure S1. Deconvoluted FTIR spectra of amide II (1500–1600 cm^−1^) and amide I (1600–1700 cm^−1^) region of processed LMMA.
