# Supplementary material for: Incorporation of Mycelium (Pleurotus eryngii) in Pea Protein Based Low Moisture Meat Analogue: Effect on Its Physicochemical, Rehydration and Structural Properties
Source: Foods. 2022 Aug 17;11(16):2476. doi: 10.3390/foods11162476 (PMC9407581; doi:10.3390/foods11162476)
Supplement: Supplementary file 1 [file foods-11-02476-s001.zip › foods-1779255-supplementary.pdf]

Supplementary Materials

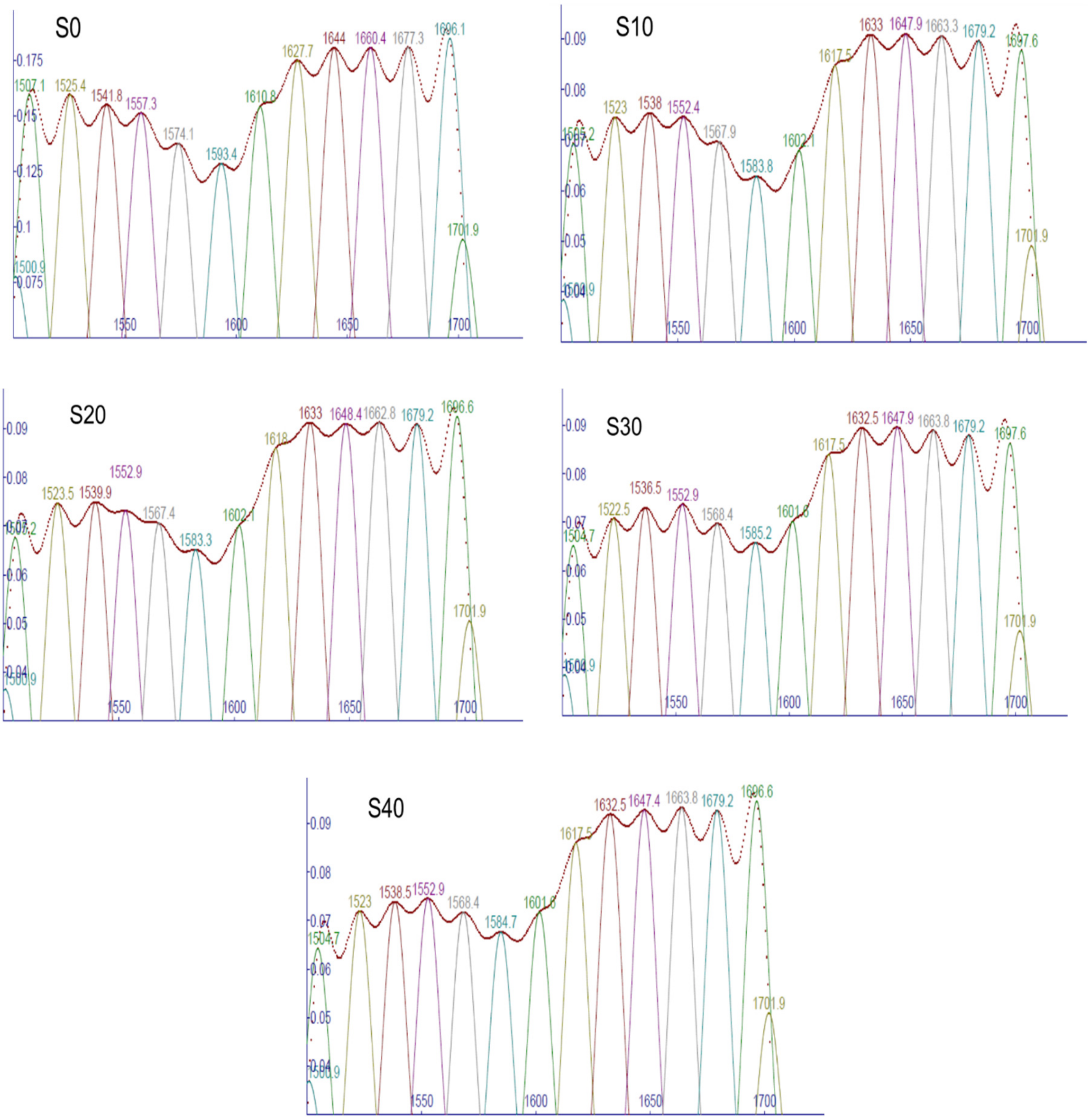

Figure S1. Deconvoluted FTIR spectra of amide II (1500-1600 cm^{-1}) and amide I (1600-1700 cm^{-1}) region of processed LMMA
